# Mr. Whitlam, on the Agaricus Campestris

**Published:** 1806-03

**Authors:** John Whitlam

**Affiliations:** Nottingham


### *To the Editors of the Medical and Physical Journal.*

GENTLEMEN,

THE fatal effects which have often been produced by eating Mushrooms, have not been sufficient to prevent their being continually used, as an article of diet. We have the evidence of some celebrated Naturalists, that what is called the esculent mushroom, when gathered from certain situations, or during some peculiar states of the atmosphere, acts as a poison.*

The disease which I am about to describe, seemed to have its origin from a source of this kind. On the 26th of last September, I was called to Mrs. F——, a married lady of about 36 years of age. She complained of pain in her throat, and had a considerable difficulty in swallowing. On inspection, I found the fauces of a dark red colour; no ulceration, nor much tumefaction. Her stomach seemed distended and loaded. She had perceived her stomach thus disordered for some days, attended with frequent and disagreeable eructations. She did not at that time recollect having eaten any thing which had particularly disagreed with her. I ordered an emetic; the volatile liniment to be applied to the external fauces; and a gargle to be used, composed of nit. potassæ; gummi mimos. nilot. et aquæ. On the 27th, the throat not quite so red: she complained of great difficulty in swallowing, which she described as proceeding from an enlargement of the root of her tongue. This part (her tongue) was moist at the edges, but the rest of it was covered with a light coloured fur. A blister was applied from ear to ear, and a gentle opening medicine was given. On the next morning her tongue was so much enlarged, that she could not speak intelliigbly; it was covered, as far as could be seen, with a very thick dark coloured substance. The difficulty of swallowing seemed now to depend altogether on the enlargement of her tongue. ℞. Infus. rosæ, quo os et fauces sæpè de die colluantur. Boracis sodæ ʒi. mel. acet. vini. aa. ℥i. misce. Her tongue was frequently rubbed with this liniment; and the opening medicine was repeated. On the 29th, the swelling had considerably abated,

---

* Nouveau Dictionnaire d'Histoire Naturelle, appliqué aux Arts, principalement à l'Agriculture & à l'Economie rurale & domestique.

abated, and she was better in every respect. Warm milk was her only diet during this treatment.

On the 3d of October, her tongue was reduced to nearly its natural size, and looked quite clean. About this time, the submaxillary glands on the right side became enlarged. The swelling extended itself down the greatest part of the neck, and she complained of great soreness about the thyroid cartilage. A saponaceous liniment was applied, and a cataplasm of the crumbs of bread and the diluted aq. lytharg. acetat. On the 6th, the soreness near the cartilage had nearly left her; but the swelling of the glands and of the neck increased, and became very painful. On the 9th, a large blister was applied to the whole swelled part; and on account of the sense of weight and distention returning in the stomach, another emetic was given. When the blister had risen, a fluid was discharged from it, having the appearance of pus; and seemed to proceed from every part where the cuticle had been detached by the blister. On account of the pain and want of sleep, an opiate was given every night; and a tonic medicine at intervals during the day, to counteract the debility attending this complaint. The discharge from the blister was kept up by a stimulating ointment; and during this time the pain considerably abated, but returned with redoubled violence when the part healed. The lower part of the tumour then increased in size: it was poulticed, and in a short time burst and discharged a large quantity of purulent matter. In the beginning of November the abscess healed, and the pain returned, extending itself to the ear on the same side.

Having observed a remission of the violent symptoms during the time when the blister was kept open, and again whilst the abscess kept discharging, I had reason to hope for permanent relief from a long continued drain from another part of the body. The event answered my expectation. A blister was applied to that part of the arm where issues are usually placed; and it was caused to discharge till the latter end of November. Another was then applied to the other arm: the pain and swelling gradually left the patient; and before Christmas, she had regained her usual state of health.

### REMARKS.

During the progress of the complaint, the patient recollected that she had eaten mushrooms on the day preceding her first indisposition. They had the appearance, as well as as the flavour of good ones, but appeared old got. She said that her pudenda et mamma had become enlarged and painful, previous to her throat being affected. After which they ceased to trouble her.

The singular symptoms attending this complaint; the parts affected in succession; the relief obtained by the discharge from the blisters, and from the abscess; afford a subject worthy of particular consideration.

The noxious effects sometimes produced by the agaricus campestris; attributed by the French Naturalists to certain changes produced on them by fogs, time of gathering them, &c. may, perhaps, in part, arise from a source which has not hitherto been explored by the Medical Chemist.

It is my intention to enter on a series of experiments, to ascertain the changes induced on mushrooms by the action of their juices on metallic substances. May not a part of their poisonous effects arise from a source of this kind? Analogy leads us to this conclusion. The solvent powers of some extremely mild substances are very evident, when brought into contact with metals. If the results of my experiments should be such as I expect, an account of them will be laid before the public.

I am, &c.

JOHN WHITLAM.

*Nottingham, Feb.* 10, 1806.

